# Alterations in intrinsic functional networks in Parkinson’s disease patients with depression: A resting‐state functional magnetic resonance imaging study

**DOI:** 10.1111/cns.13467

**Published:** 2020-10-21

**Authors:** Yi‐Hui Qiu, Zhi‐Heng Huang, Yu‐Yuan Gao, Shu‐Jun Feng, Biao Huang, Wan‐Yi Wang, Qi‐Huan Xu, Jie‐Hao Zhao, Yu‐Hu Zhang, Li‐Min Wang, Kun Nie, Li‐Juan Wang

**Affiliations:** ^1^ Department of Neurology Guangdong Provincial Peoples' Hospital Guangdong Academy of Medical Sciences Guangdong Neuroscience Institute Guangzhou China; ^2^ Department of Radiology Guangdong Provincial Peoples' Hospital Guangdong Academy of Medical Sciences Guangzhou China

**Keywords:** depression, functional magnetic resonance imaging, graph theory, network‐based statistical analysis, Parkinson’s disease

## Abstract

**Aims:**

The aim of this research was to investigate the alterations in functional brain networks and to assess the relationship between depressive impairment and topological network changes in Parkinson's disease (PD) patients with depression (DPD).

**Methods:**

Twenty‐two DPD patients, 23 PD patients without depression (NDPD), and 25 matched healthy controls (HCs) were enrolled. All participants were examined by resting‐state functional magnetic resonance imaging scans. Graph theoretical analysis and network‐based statistic methods were used to analyze brain network topological properties and abnormal subnetworks, respectively.

**Results:**

The DPD group showed significantly decreased local efficiency compared with the HC group (*P* = .008, FDR corrected). In nodal metrics analyses, the degree of the right inferior occipital gyrus (*P* = .0001, FDR corrected) was positively correlated with the Hamilton Depression Rating Scale scores in the DPD group. Meanwhile, the temporal visual cortex, including the bilateral middle temporal gyri and right inferior temporal gyrus in the HC and NDPD groups and the left posterior cingulate gyrus in the NDPD group, was defined as hub region, but not in the DPD group. Compared with the HC group, the DPD group had extensive weakening of connections between the temporal‐occipital visual cortex and the prefrontal‐limbic network.

**Conclusions:**

These results suggest that PD depression is associated with disruptions in the topological organization of functional brain networks, mainly involved the temporal‐occipital visual cortex and the posterior cingulate gyrus and may advance our current understanding of the pathophysiological mechanisms underlying DPD.

## Graphical abstract

The functional brain networks of the patients with Parkinson's disease (PD) with depression exhibite small‐world architecture. PD depression is associated with disruptions in the topological organization of functional brain networks, mainly involved the temporal‐occipital visual cortex and the posterior cingulate gyrus.

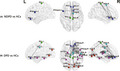

## 1 INTRODUCTION

Parkinson's disease (PD) is a common neurodegenerative disease with a growing incidence in recent decades. Depression is considered one of the most common nonmotor symptoms of PD, with an incidence of up to 40%.^1^ In addition, depression is one of the established clinical prodromal markers and may be an independent risk factor for PD,^2^, ^3^ often occurring before motor symptoms appear and causing a serious negative impact on PD patients' quality of life.^4^ However, the pathophysiology underlying the contribution of depression to PD remains unclear. Thus, exploring the pathological mechanisms of depression in PD may contribute to early identification and effective treatment of PD patients in the clinical prodromal stage of the disease; that is, it might delay the progression of the disease and improve quality of life.

With the rapid development of imaging technology and its continuous application in neuroscience research, strong noninvasive technical support is available for exploring the pathogenesis of neuropsychiatric diseases and realizing the early diagnosis of PD patients with depression (DPD). Graph theory provides a powerful mathematical framework for describing the topological organization of brain networks in terms of nodes (ie, brain regions) and edges (ie, physical or functional connections between brain regions).^5^ The latest research has suggested that network topological metric measurements derived using graph theory may be potential biomarkers in PD to evaluate disease progression and to monitor therapeutic effects.^6^ By using diffusion tensor imaging, one study showed that despite preserved small‐world topology, DPD exhibited higher network efficiency in the fronto‐limbic system compared to that in PD patients without depression (NDPD).^7^ Gou et al,^8^ by using graph theory and network‐based statistic (NBS) methods, showed that the integration of the structural brain network was impaired with disrupted connectivity of the limbic and visual systems in de novo and drug‐naïve DPD patients. Notably, these results were approximately consistent with those obtained from depressive patients, suggesting that deficits of the regional and connectivity characteristics in the structural networks were primarily found in the frontal brain regions, limbic system, and occipital lobe compared to that in healthy controls (HCs).^9^, ^10^ Therefore, it is an urgent problem to explore the characteristic imaging markers of DPD and to realize early diagnosis and treatment. A previous study showed that even though the overall structural organization of the PD connectome remains robust at relatively early disease stages, there is a breakdown in the functional modular organization of the PD connectome.^11^ Therefore, characterization of the functional network connectome in DPD patients may help researchers to further explore the mechanisms underlying DPD.

The purpose of this study was to investigate the changes in the topological properties and the functional connectivity of the whole‐brain functional network in patients with DPD and to assess whether depressive impairment is correlated with functional topological network changes in DPD patients.

## 2 MATERIALS AND METHODS

### 2.1 Participants

Forty‐five patients with idiopathic PD and 27 age‐, sex‐, and years of education–matched HCs were enrolled from the Department of Neurology at Guangdong Provincial Peoples' Hospital in China. PD was diagnosed according to the Movement Disorder Society (MDS) Clinical Diagnostic Criteria for PD. Twenty‐two PD patients were diagnosed with depressive disorder according to the Diagnostic and Statistical Manual of Mental Disorders, 5th edition (DSM‐V) criteria,^12^, ^13^ and the remaining twenty‐three patients had PD alone. None of the patients had used any antidepressant(s). Individuals with Parkinson's plus syndrome (PPS) caused by vascular factors, toxins, drugs, etc, were excluded. Participants were also excluded if they had dementia, other severe neuropsychiatric disorders, or serious respiratory, cardiovascular, and digestive diseases. All participants were right‐handed. This study was approved by the Research Ethics Committee of Guangdong Provincial People's Hospital, Guangdong Academy of Medical Sciences. All participants provided written informed consent prior to their inclusion in the study.

### 2.2 Neuropsychological assessments

Motor symptom severity in PD patients was measured by the MDS Unified PD Rating Scale (MDS‐UPDRS) part III and the Hoehn and Yahr (H&Y) scale. All participants were screened for depression, anxiety, and cognitive function using the Hamilton Depression Rating Scale (HAMD), Hamilton Anxiety Rating Scale (HAMA), and Mini‐Mental State Examination (MMSE), respectively. The levodopa equivalent dose (LED) was calculated for each PD patient.

### 2.3 MRI data acquisition

All the PD subjects in this study underwent rs‐fMRI after discontinuing antiparkinsonian medication for more than 12 hours. The scans were performed on a 3.0T GE SIGNA MR scanner (Signa Excite HD; GE Healthcare) with an 8‐channel head coil in the Department of Radiology of Guangdong Provincial People's Hospital. All participants were required to keep their eyes closed, relax, and remain awake during the MRI scans. The rs‐fMRI scanning parameters were as follows: a gradient‐echo echo‐planar imaging (GRE‐EPI) sequence with a repetition time (TR)/echo time (TE) = 2000/30 ms, matrix = 64 × 64, 30 axial slices covering the entire brain, field of view = 240 × 240 mm, slice thickness = 4 mm, interslice space = 1 mm, NEX = 1, voxel size = 3.75 mm × 3.75 mm × 4 mm, and time points = 186. Axial scans were parallel to the anterior‐posterior commissure (AC‐PC) line. High‐resolution 3D T1‐weighted anatomical images were obtained for coregistration with the functional data. A fast spoiled gradient recalled echo inversion recovery sequence was used to acquire sagittal T1‐weighted images with the following parameters: TR/TE = 8.4/3.3 ms, matrix = 256 × 256, flip angle = 13°, slice thickness = 1 mm, and voxel size = 0.94 mm × 0.94 mm × 1 mm.

### 2.4 Data preprocessing

Data preprocessing was performed on the MATLAB‐based DPABI, the Data Processing Assistant for rs‐fMRI. The first ten volumes of the functional images were discarded. The remaining volumes were slice‐time corrected, and realignment was performed to correct the motion between time points. Head motion parameters were computed by estimating the translation in each direction and the angular rotation on each axis for each volume. The subjects with motion (a mean framewise displacement [FD] Jenkinson^14^) greater than 2*standard deviations (SDs) above the group mean motion were excluded.^15^ According to this exclusion criterion, only two subjects were excluded from the HC group (Tables S1 and S2). No significant intergroup differences were found in head motion (Table 1). The fMRI data were coregistered to the same subject's high‐resolution T1‐weighted image. The coregistered images were segmented into gray matter, white matter (WM), and cerebrospinal fluid (CSF) and were then spatially normalized to the standard stereotaxic coordinates of the Montreal Neurological Institute space using an echo‐planar imaging template and resampled into a voxel size of 3*3*3 mm^3^. Then, WM, CSF, and head motion were removed as nuisance variables; head motion was removed using the Friston 24‐parameter model. The generated images were spatially smoothed with a 4‐mm full‐width at half‐maximum Gaussian kernel. A temporal filter (0.01‐0.1 Hz) was used to decrease the effect of low‐frequency drifts and physiological high‐frequency noise. In addition, adequate quality control was conducted on MRI data, which is essential for establishing reliable results.^16^

**Table 1:** Demographic and clinical characteristics and global topological properties of the NDPD, DPD, and HC groups

Measure	NDPD	DPD	HCs	F/H/χ^2^/Z	*P*‐value	*P*‐value (NDPD vs HCs)	*P*‐value (DPD vs HCs)	*P*‐value (DPD vs NDPD)
Sex (male/female)	16/7	13/9	9/16	5.737	0.057			
Age	66.00 (23.00)	64.50 (17.00)	55.00 (11.50)	1.273	0.529			
Education (y)	10.09 ± 4.842	9.50 ± 4.718	8.80 ± 3.764	0.505	0.606			
Course of the disease (y)	3.00 (4.50)	3.00 (4.50)	NA	−0.820	0.412			
LED	0.00 (275.00) [0.00,675.00]	0.00 (505.00) [0.00,1050.00]	NA	−1.269	0.204			
H&Y Stage	2.00 (1.00)	2.00 (1.50)	NA	−0.074	0.941			
MDS‐UPDRS‐III	35.00 (9.00)	39.00 (17.25）	NA	−1.957	0.050			
MMSE	28.00 (2.00)	28.00 (3.00)	27.50 (4.00)	0.973	0.615			
HAMD	7.00 (4.00)	22.50 (9.25)	1.00 (3.50)	53.621	<0.001	0.009	<0.001	<0.001
HAMA	6.00 (4.00)	18.00 (11.50)	2.00 (2.00)	49.093	<0.001	0.014	<0.001	<0.001
Mean FD_Jenkinson	0.067(0.037)	0.054(0.091)	0.065(0.055)	0.068	0.967			
Eglob	0.260 ± 0.005	0.257 ± 0.009	0.258 ± 0.008	0.883	0.418			
Eloc	0.345 ± 0.012	0.341 ± 0.011	0.348 ± 0.008	3.230	0.046	0.193	0.008	0.266
Modularity	0.143 ± 0.017	0.140 ± 0.018	0.141 ± 0.021	0.115	0.892			
Lp	0.842 ± 0.031	0.858 ± 0.047	0.862 ± 0.070	0.917	0.405			
Cp	0.271 ± 0.019	0.267 ± 0.020	0.278 ± 0.011	2.437	0.095			
λ	0.487 ± 0.012	0.491 ± 0.016	0.494 ± 0.017	1.394	0.255			
γ	0.868 ± 0.098	0.858 ± 0.113	0.901 ± 0.154	0.765	0.469			
σ	0.778 ± 0.084	0.765 ± 0.102	0.796 ± 0.137	0.491	0.614			

Abbreviations: Cp, the clustering coefficient; DPD, Parkinson's disease patients with depression; Eglob, the global efficiency; Eloc, the local efficiency; H&Y, Hoehn and Yahr scale; HAMA, Hamilton Anxiety Rating Scale; HAMD, Hamilton Depression Rating Scale; HCs, healthy controls; LED, levodopa equivalent dose; Lp, the characteristic path length; MDS‐UPDRS‐III, MDS Unified Parkinson's Disease Rating Scale (MDS‐UPDRS) part III; MMSE, Mini‐Mental State Examination; NA, not applicable; NDPD, Parkinson's disease patients without depression; γ, the normalized clustering coefficient; λ, the normalized characteristic path length; σ, small worldness.

### 2.5 Brain network construction

The binarized functional brain network was constructed by GRETNA,^17^ a graph theoretical network analysis toolbox for imaging connectomics. The functional connectivity matrices (composed of positive correlations), which were used to analyze brain network topological properties and to examine abnormal subnetworks, were constructed in two major steps: network node definition and network edge definition. The atlas of automated anatomical labeling (AAL)^18^ was employed to parcellate the entire brain into 90 (45 for each hemisphere) cortical and subcortical regions of interest (Table 2), with each representing a node of the network. Pearson's correlation coefficients for each pair of regions were computed for the mean time series for each of the 90 regions and defined as the edge of the network. Then, Fisher's *z* transformation was performed to transform the data into *z*‐values that were close to normally distributed. Sparsity (Sp) was employed as the threshold metric to compute the network properties, with a range of 0.05~0.50 and a step length of 0.01.^19^, ^20^ Sparsity is defined as the ratio of the number of actual edges divided by the maximum possible number of edges in a network. For networks with the same number of nodes, the sparsity threshold ensures the same number of edges for each network by applying a subject‐specific connectivity strength threshold and therefore allowing examination of the relative network organization.^21^

**Table 2:** The abbreviations of the 90 brain regions in AAL‐90 atlas

Number	Region	Abbreviations
1, 2	Precentral gyrus	PreCG
3, 4	Superior frontal gyrus, dorsolateral	SFGdor
5, 6	Superior frontal gyrus, orbital part	ORBsup
7, 8	Middle frontal gyrus	MFG
9, 10	Middle frontal gyrus, orbital part	ORBmid
11, 12	Inferior frontal gyrus, opercular part	IFGoperc
13, 14	Inferior frontal gyrus, triangular part	IFGtriang
15 ,16	Inferior frontal gyrus, orbital part	ORBinf
17, 18	Rolandic operculum	ROL
19, 20	Supplementary motor area	SMA
21, 22	Olfactory cortex	OLF
23, 24	Superior frontal gyrus, medial	SFGmed
25, 26	Superior frontal gyrus, medial orbital	ORBMed
27, 28	Gyrus rectus	REC
29, 30	Insula	INS
31, 32	Anterior cingulate and paracingulate gyri	ACG
33, 34	Median cingulate and paracingulate gyri	MCG
35, 36	Posterior cingulate gyrus	PCG
37, 38	Hippocampus	HIP
39, 40	Para hippocampal gyrus	PHG
41, 42	Amygdala	AMYG
43, 44	Calcarine fissure and surrounding cortex	CAL
45, 46	Cuneus	CUN
47, 48	Lingual gyrus	LING
49, 50	Superior occipital gyrus	SOG
51, 52	Middle occipital gyrus	MOG
53, 54	Inferior occipital gyrus	IOG
55, 56	Fusiform gyrus	FFG
57, 58	Postcentral gyrus	PoCG
59, 60	Superior parietal gyrus	SPG
61, 62	Inferior parietal lobule	IPL
63, 64	Supramarginal gyrus	SMG
65, 66	Angular gyrus	ANG
67, 68	Precuneus	PCUN
69, 70	Paracentral lobule	PCL
71, 72	Caudate	CAU
73, 74	Putamen	PUT
75, 76	Pallidum	PAL
77, 78	Thalamus	THA
79, 80	Heschl gyrus	HES
81, 82	Superior temporal gyrus	STG
83, 84	Temporal pole: superior temporal gyrus	TPOsup
85, 86	Middle temporal gyrus	MTG
87,88	Temporal pole: middle temporal gyrus	TPOmid
89, 90	Inferior temporal	ITG

### 2.6 Functional brain network analyses

Graph theory was applied to analyze the functional brain network. For the global network, the global efficiency (Eglob) and characteristic path length (Lp) were used to investigate the integration of the functional brain network, while segregation was assessed by the local efficiency (Eloc), clustering coefficient (Cp), and modularity. For each participant, to assess whether the network had small‐world properties, the network measures were normalized to comparable values from random networks (N = 100). Small‐world organization was assessed by the normalized Lp (λ), the normalized Cp (γ), and small worldness (σ). The local topological properties (the degree, betweenness centrality, and nodal efficiency) were obtained to measure the regional network organization. To investigate group differences in these networks, we calculated the area under the curve for each network metric, which provides a summarized scalar for topological characterization of brain networks independent of specific threshold selection. The NBS approach, which is a validated, nonparametric statistical approach for controlling familywise errors in connectome analyses,^22^ was utilized to further identify functional connections showing differences between each pair of groups.

### 2.7 Statistical analysis

All analyses of demographic and clinical characteristics were performed using SPSS Statistics 22.0. The χ^2^ test was used to analyze the sex ratios among the three groups. One‐way analysis of variance (ANOVA) was used for education years, and a pairwise post hoc test was subsequently used to identify significant main group differences with correction by the Bonferroni test. Age and MMSE, HAMD, and HAMA scores were compared by the Kruskal‐Wallis H test and further evaluated by post hoc tests (Bonferroni's test). The Mann‐Whitney *U* test was used to compare the LED, H&Y stage, and MDS‐UPDRS‐III scores between the DPD and NDPD groups. The statistical significance threshold was set at *P* < .05.

The imaging data were analyzed based on a general linear model (GLM) on GRETNA. ANOVA was used to compare the AUCs of network topological properties among the three groups. Post hoc analyses were performed using two‐sample *t* tests in a pairwise manner within the areas identified by the ANOVA. With age, years of education, disease course, the LED, H&Y stage, and MDS‐UPDRS‐III, MMSE, and HAMA scores as covariates, correlation analysis was used to test correlations between network topological properties and HAMD scores in the DPD group. To address the problem of multiple comparisons in the nodal metrics, a false discovery rate (FDR) *q* of 0.05 multiple comparisons correction was performed.

Next, to determine the significance levels of altered connectivity networks in NBS analysis, we first performed two‐sample *t* test at each edge independently to test for significant differences in the value of connectivity between two groups. A primary component‐forming threshold (*P* = .05) was then applied to form a set of suprathreshold edges among which any connected components and their size could then be determined. Next, the statistical significance of the size of each observed component was then evaluated with respect to an empirical null distribution of maximal component sizes obtained under the null hypothesis of random group membership (50,000 permutations). Subnetworks that were significant at a corrected level of *P* = .001 were reported. The effects of age, gender, and years of education were adjusted for these analyses.

## 3 RESULTS

### 3.1 Population characteristics

The demographic and clinical features of the participants are presented in Table 1. No significant differences were observed in age, sex, years of education, or MMSE scores among the three groups. In addition, the DPD and NDPD patients had comparable values for disease course, the LED, H&Y stage, and MDS‐UPDRS III scores. However, both the HAMD and HAMA scores in the DPD group were significantly higher than those in the other two groups (*P* < .001), which is consistent with previous studies revealing that DPD coexisted with anxiety disorder.

### 3.2 Global network analyses

**Figure 1:**
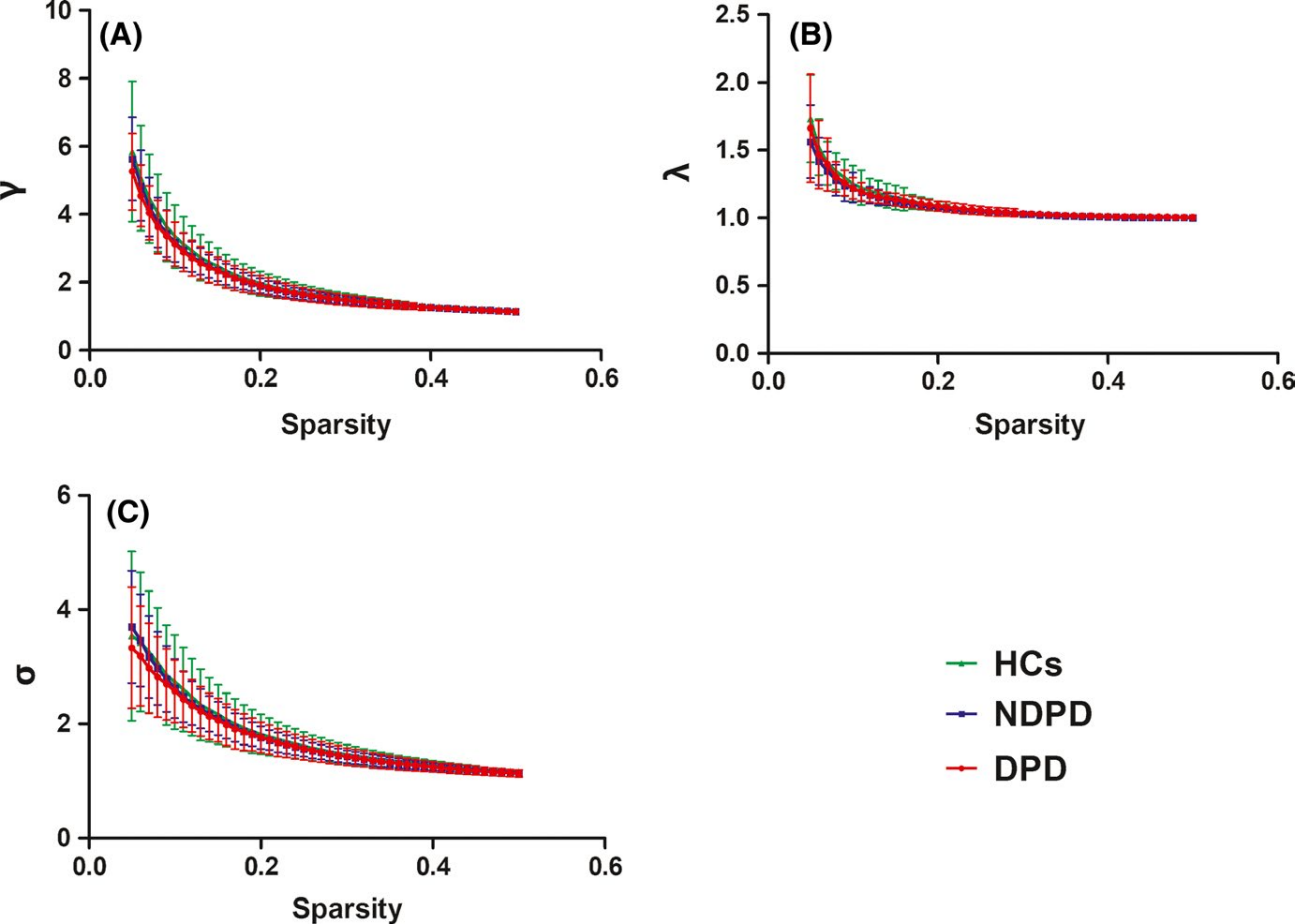
The results of small‐world properties of the three groups of subjects under different sparsity. Bars and error bars represent mean values and standard deviation, respectively. λ, the normalized characteristic path length; γ, the normalized clustering coefficient; σ, small worldness; DPD, Parkinson's disease patients with depression; NDPD, Parkinson's disease patients without depression; HCs, healthy controls [Colour figure can be viewed at wileyonlinelibrary.com]

Over the sparsity range of 0.05‐0.50 (step = 0.01), the DPD, NDPD, and HC groups exhibited a high‐efficiency small‐world topology (γ = Cp/Cr > 1, λ = Lp/Lr ≈ 1 and σ = γ/λ > 1; Figure 1).

Statistical comparisons were performed to detect significant differences in the AUCs of global parameters among the three groups. The DPD group showed a significantly lower integrated Eloc (*P* = .008, FDR corrected) compared with the HC group. No significant differences (*P* > .05) were found in Eglob, Lp, Cp, modularity, γ, λ, and σ (Table 1).

### 3.3 Regional network analyses

#### 3.3.1 Intergroup differences in local topological properties

Brain regions exhibiting significant intergroup differences in at least one nodal metric (the degree, efficiency, or betweenness) were identified. But there were no brain regions surviving the FDR correction for multiple comparisons.

#### 3.3.2 Differences in hub regions among the three groups

The hubs were identified as nodes with both degree and betweenness centrality that were one SD above the network averages.^23^ In the HC group, five hub regions were defined in the temporal visual area, including the bilateral fusiform gyri (FFG), bilateral middle temporal gyri (MTG), and right inferior temporal gyrus (ITG.R). Compared with the HC group, the NDPD group lacked the bilateral FFG as hub regions, while the left posterior cingulate gyrus (PCG.L) was a hub region. However, no hub regions were screened out in the DPD group (Tables 3 and S5).

**Table 3:** Hub regions in the HC, NDPD and DPD groups

Groups	Region	Degree	Betweenness
HC	ITG.R	17.195	61.361
	MTG.L	16.853	48.454
	MTG.R	16.564	44.172
	FFG.L	16.630	45.940
	FFG.R	16.826	54.120
NDPD	ITG.R	17.195	61.361
	MTG.L	16.853	48.454
	MTG.R	16.564	44.172
	PCG.L	16.039	46.224
DPD	NA	NA	NA

Note

The abbreviations of the 90 brain regions are given in Table 2.

Abbreviations: DPD, Parkinson's disease patients with depression; HC, healthy control; L, left hemisphere; NA, not applicable; NDPD, Parkinson's disease patients without depression; R, right hemisphere.

### 3.4 Correlations between network topological properties and HAMD scores in the DPD group

**Figure 2:**
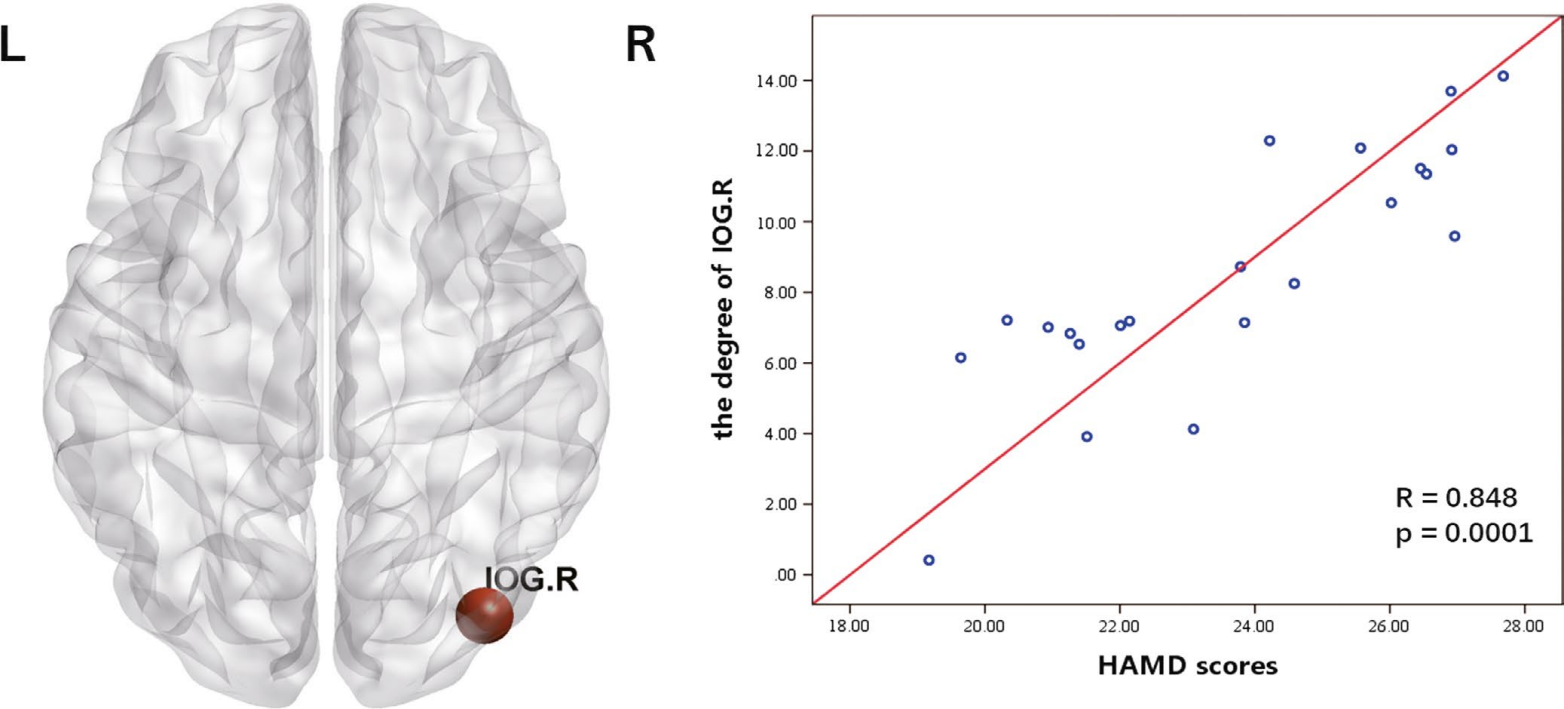
The results of the correlation analysis between the degree and HAMD scores in the DPD group. The abbreviations of the 90 brain regions are given in Table 2. L, left hemisphere; R, right hemisphere; DPD, Parkinson's disease patients with depression [Colour figure can be viewed at wileyonlinelibrary.com]

Figure 2 and Table S3 present the results of the correlation analysis between the degree and HAMD scores in the DPD group. A significantly and specifically positive correlation was observed in the right inferior occipital gyrus (IOG.R; *r* = .848, *P* = .0001, FDR corrected) that was not present in the NDPD group (Table S4). There was no correlation between HAMD scores and the other network topological properties surviving the FDR correction for multiple comparisons.

### 3.5 Functional connectivity characteristics

**Figure 3:**
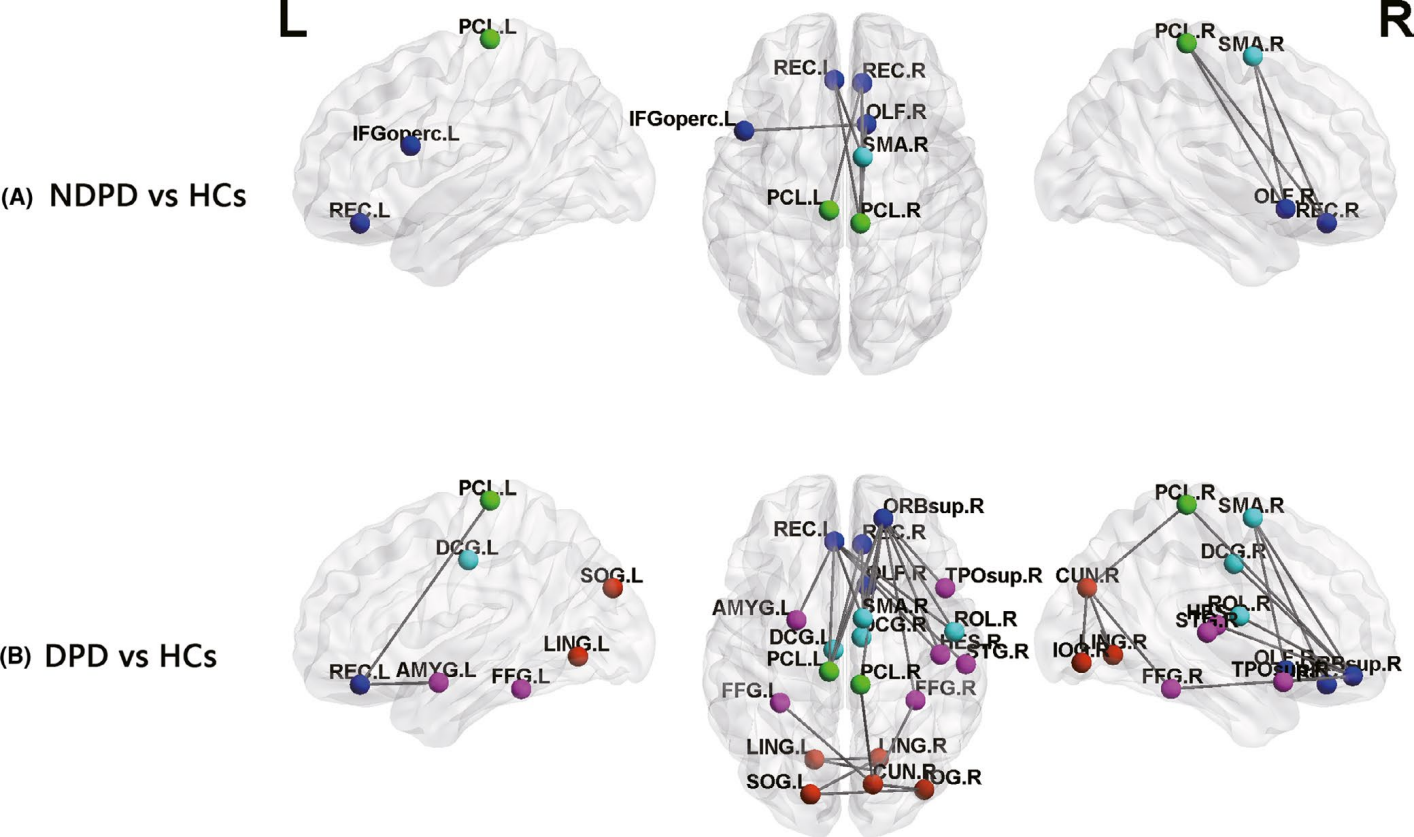
A, The abnormal subnetworks constituted in NDPD group compared with HC group; B, the abnormal subnetworks constituted in DPD group compared with HC group. There were no significant differences between NDPD compared with DPD. Light blue, the nodes in the frontal lobe; blue, the nodes in the prefrontal lobe; green, the nodes in the parietal lobe; purple, the nodes in the temporal lobe; red, the nodes in the occipital lobe. The abbreviations of the 90 brain regions are given in Table 2. L, left hemisphere; R, right hemisphere. DPD, Parkinson's disease patients with depression; NDPD, Parkinson's disease patients without depression; HC, healthy control [Colour figure can be viewed at wileyonlinelibrary.com]

In the NBS analysis, the primary test statistic thresholds were set to 3.521 (DPD vs HCs), 3.533 (NDPD vs HCs), and 2.019 (DPD vs NDPD) according to the test results. One subnetwork consisting of seven nodes and eight edges with decreased functional connectivity was characterized in the NDPD group compared with the HC group, including the left inferior frontal gyrus, opercular part (IFGoperc.L), right olfactory cortex (OLF.R), bilateral gyrus rectus (REC), right supplementary motor area (SMA.R), and bilateral paracentral lobule (PCL; Figure 3, Table S6). Compared with the HC group, we localized a connected subnetwork involving more extensive brain regions with 21 nodes and 29 edges that were significantly decreased in the DPD group, which contained an extensive portion of the prefrontal (eg, the bilateral median cingulate and paracingulate gyri) and temporal‐occipital (eg, the amygdala, bilateral FFG, and occipital lobe) lobes in addition to the same brain regions included in the different subnetwork between the NDPD and HC groups (Figure 3, Table S7). Most of these regions were the components of the prefrontal‐limbic network and temporal‐occipital visual cortex. In contrast, the DPD group did not show any alterations in functional connectivity compared with the NDPD group.

## 4 DISCUSSION

In the present study, we comprehensively examined whole‐brain resting‐state networks for functional changes in DPD patients using graph theoretical and network‐based analyses. Our findings revealed that the brain functional network of DPD patients preserved the small‐world property, but the Eloc was significantly reduced compared with the HCs. In nodal metrics analyses, the degree of the occipital visual cortex in DPD group was found to be positively associated with the depressive symptoms. Meanwhile, there was a reorganization of the network's hubs in the temporal visual cortex and PCG.L in the DPD group. Moreover, different functional connectivity trends were observed in the NDPD and DPD groups vs the HC group.

A small‐world network involves a combination of a high Cp (a measure of local network connectivity) and a short characteristic Lp (a measure of global network connectivity), reflecting a highly effective topological organization combining regional specialization and efficient global information transfer to realize efficient transmission of information.^24^, ^25^, ^26^ Here, we found that the functional brain networks of the DPD, NDPD and HC individuals exhibited a small‐world architecture, which is consistent with previous findings suggesting the preservation of a small‐world architecture in the presence of pathology.^21^ Despite the common small‐world topology, the Eloc showed significantly smaller values over a wide range of sparsity in the DPD patients relative to the HC subjects. Given that the small‐world model reflects an optimal balance between local specialization and global integration, these results indicate a disturbance of the normal balance in functional brain networks of DPD patients. This disturbance can be attributed to the altered nodal characteristics and decreased functional connections in DPD patients. However, this significant difference did not survive between DPD vs NDPD groups. At the same time, our findings are not consistent with previous research reporting that small‐world properties in PD patients differed from those in HCs, such as decreased Cp or increased Lp.^27^, ^28^ These may be related to the effects of dopaminergic drugs. Functional magnetic resonance studies have revealed that degeneration of nigrostriatal neurons in PD may be associated with large‐scale network reorganization and that levodopa tended to normalize the disrupted network topology in PD patients.^29^ Although all PD subjects underwent rs‐fMRI after discontinuing antiparkinsonian medication for more than 12 hours, the interference of drugs could not be completely excluded.

The nodal degree is the sum of all binary/weighted edges of one node, which measures the single nodal connectivity to the rest of the nodes in a network. Regarding the significantly positive correlation between depressive symptoms and the betweenness centrality of IOG.R, which is the striate cortex regions of the visual cortex, previous studies have reported conclusions consistent with our findings. Some rs‐fMRI studies have shown that the value of regional homogeneity in the occipital lobe was decreased^30^ and that the synchrony of interhemispheric resting‐state functional connectivity was impaired in the occipital lobe in DPD patients.^31^ In particular, a graph theory study showed that the node topological properties of the bilateral lingual and bilateral inferior occipital regions were significantly associated with Geriatric Depressive Scale (GDS) scores in DPD patients.^8^ Moreover, studies also found that patients with DPD exhibited hyperperfusion and accelerated brainwave activity in the occipital cortex.^32^, ^33^ Kim et al^32^ discovered that perfusion in the occipital lobe was increased and regional cerebral blood flow (rCBF) in the occipital cluster was positively related to the GDS scores in DPD patients. A quantitative electroencephalogram study showed the higher amplitude in beta in occipital lobe areas in rapid eye moment relative to nonrapid eye moment 2 were significantly different in DPD and NDPD patients.^33^ Increased cerebral blood flow and brainwave activity in these areas may accelerate effective information transmission. The mechanism remains unclear but may be related to the reduced γ‐aminobutyric acid (GABA) levels, while GABAergic neurons are mostly distributed in the occipital cortex of depressed patients. Prior research has suggested that occipital levels of GABA were significantly lower in recovered depressed patients than in healthy controls.^34^ Inverse correlations between the GABA levels and rCBF in the brain have also been suggested by the previous literature.^35^, ^36^ Further study revealed that increased baseline activation in the occipital cortex predicted antidepressant response.^37^ Accordingly, we considered that changes of topological attributes in occipital visual regions may serve as useful targets for biomarkers in assessing the severity and progression of DPD.

Additionally, the extrastriate cortex regions of the visual cortex (bilateral MTG and ITG.R) were identified as network hubs in the HC and NDPD groups, but not in the DPD group, which were consistent with a recent MRI study reporting that the node topological properties of the temporal visual area were significantly associated with depressive symptoms in PD patients.^8^ Previous studies have shown that neurodegenerative diseases target brain regions that are especially highly correlated in healthy subjects. These regions are the network hubs, which play a crucial role in the network, as they interact with many brain regions.^38^ One study showed that depression was correlated with impaired color vision in PD patients through clinical observation and inferred that the visual system is crucial in the depressive pathology in PD patients.^39^ We speculate that PD depression might disrupt the visual functional network and reorganize the network's hubs.

In NBS analysis, compared with HCs, the functional connections between the regions of the temporal‐occipital visual cortex and the prefrontal‐limbic network were significantly weakened in DPD patients, which did not appear in the comparison between NDPD patients and HCs. The most common cognitive feature of depression is a processing bias toward negatively affective stimuli, which is associated with brain activation in visual areas involved in early visual processing of affective stimuli, limbic and paralimbic regions involved in evaluating and integrating sensory and affective information, and prefrontal areas involved in top‐down emotion regulation.^40^ Combining our findings and those of previous studies, we propose that visual cortex dysfunction may play an important role in the pathophysiology of DPD and impact the connections with the brain regions involved in cognition and emotion, ultimately causing individuals to selectively attend to negative information and experience difficulty disengaging attention from negative stimuli. These attention biases subsequently reinforce a sad mood and contribute to a persistent depressive episode. Although no results showed significance between the DPD and NDPD groups under NBS correction, the different trends observed provided evidence that advances our understanding of the pathophysiological mechanism of DPD.

In addition, the PCG was one of the network hubs in the NDPD group, but not in the DPD group. The cingulate gyrus is an important area where the frontal cortex, insula, amygdala, and hypothalamus interconnect. As a part of the limbic system, it participates in emotion regulation, cognitive function and self‐control. Posterior cingulate cortex (PCC) represents a core hub for the posterior subnetwork of the default mode network and plays a key role in integrating self‐relative information, retrieval of episodic memory, and autobiographical search, the impairment of which contributes to the characteristic symptom of self‐focused rumination and plays a part in the pathogenesis of primary depression.^41^, ^42^ Gao et al^43^ found that patients with first‐episode treatment‐naive depression showed increased network homogeneity in the PCC. One study in unmedicated major depressive disorder patients reported higher functional connectivities between the PCC and lateral orbitofrontal cortex and between the angular and middle frontal gyri.^44^ A previous study also observed that functional connectivity in the right PCC was increased in DPD patients and negatively correlated with depression scores.^45^ Given these findings, we speculate that DPD and primary depression may share a similar pathological change and that the PCC plays a key hub role in the pathogenesis of primary depression, as well as in DPD patients.

Some limitations of our study should be noted. First, most of the subjects had regularly taken antiparkinsonian drugs, and the interference of drugs on the results was not completely eliminated. Second, this study had a cross‐sectional design and a relatively small sample size. Third, although the AAL atlas is still widely applied to investigate human brain organizations, a previous study suggested that the topological organization of brain networks was affected by various parcellation strategies.^46^ Furthermore, this study adopted a single imaging technology to only preliminarily explore the possible relationship between changes in the brain functional network and DPD. Therefore, longitudinal follow‐up observations are needed, drug‐naïve PD patients should be recruited, and different parcellation schemes should be used in conjunction in future large‐scale studies using multimodal MRI techniques to further explore the pathogenesis of DPD.

In conclusion, our study explored the functional brain network in DPD patients based on rs‐fMRI. Our results revealed that PD depression is associated with disruptions in the topological organization of functional brain networks, mainly involved the temporal‐occipital visual cortex and the posterior cingulate gyrus. These findings may advance our current understanding of pathophysiological mechanism underlying DPD.

## CONFLICT OF INTEREST

The authors have no conflict of interest to disclose.

## Supporting information

Tables S1‐S7

## Data Availability

The data that support the findings of this study are available in the supplementary material of this article.
