# Supplementary material for: Alterations in intrinsic functional networks in Parkinson’s disease patients with depression: A resting‐state functional magnetic resonance imaging study
Source: CNS Neurosci Ther. 2020 Oct 21;27(3):289–98. doi: 10.1111/cns.13467 (PMC7871794; doi:10.1111/cns.13467)
Supplement: Supplementary file 1 — Tables S1‐S7 [file CNS-27-289-s001.docx]

**Supplementary Table 1 the Mean FD_Jenkinson of each subject in DPD, NDPD and HC groups**

| Group | Subject | Mean FD_Jenkinson | Group | Subject | Mean FD_Jenkinson | Group | Subject | Mean FD_Jenkinson |
| --- | --- | --- | --- | --- | --- | --- | --- | --- |
| DPD | 1 | 0.04770139 | NDPD | 1 | 0.08182405 | HCs | 1 | 0.0521078 |
|  | 2 | 0.03044417 |  | 2 | 0.04569094 |  | 2 | 0.087004 |
|  | 3 | 0.0470675 |  | 3 | 0.06235687 |  | 3 | 0.06950913 |
|  | 4 | 0.07986195 |  | 4 | 0.08960796 |  | 4 | 0.2323614 |
|  | 5 | 0.05007433 |  | 5 | 0.1155302 |  | 5 | 0.04160502 |
|  | 6 | 0.05527412 |  | 6 | 0.03962838 |  | 6 | 0.04802887 |
|  | 7 | 0.125673 |  | 7 | 0.09242557 |  | 7 | 0.06464495 |
|  | 8 | 0.0470937 |  | 8 | 0.04002859 |  | 8 | 0.0738563 |
|  | 9 | 0.06204918 |  | 9 | 0.09036702 |  | 9 | 0.03914313 |
|  | 10 | 0.1427681 |  | 10 | 0.05327989 |  | 10 | 0.2209448 |
|  | 11 | 0.162989 |  | 11 | 0.08288213 |  | 11 | 0.1381814 |
|  | 12 | 0.1496482 |  | 12 | 0.04258584 |  | 12 | 0.05403091 |
|  | 13 | 0.1349846 |  | 13 | 0.08301437 |  | 13 | 0.04437671 |
|  | 14 | 0.06433388 |  | 14 | 0.1140915 |  | 14 | 0.03419649 |
|  | 15 | 0.05313776 |  | 15 | 0.04621609 |  | 15 | 0.04700057 |
|  | 16 | 0.1453892 |  | 16 | 0.06575899 |  | 16 | 0.122092 |
|  | 17 | 0.03759015 |  | 17 | 0.06719332 |  | 17 | 0.1064687 |
|  | 18 | 0.1536904 |  | 18 | 0.05517537 |  | 18 | 0.1085335 |
|  | 19 | 0.04739014 |  | 19 | 0.05294903 |  | 19 | 0.06024495 |
|  | 20 | 0.04412338 |  | 20 | 0.0529736 |  | 20 | 0.09935729 |
|  | 21 | 0.02509936 |  | 21 | 0.08109869 |  | 21 | 0.07745636 |
|  | 22 | 0.04066889 |  | 22 | 0.1022352 |  | 22 | 0.05813459 |
|  |  |  |  | 23 | 0.07421314 |  | 23 | 0.0356823 |
|  |  |  |  |  |  |  | 24 | 0.1236743 |
|  |  |  |  |  |  |  | 25 | 0.07079253 |
|  |  |  |  |  |  |  | 26 | 0.1336291 |
|  |  |  |  |  |  |  | 27 | 0.05441586 |

DPD, Parkinson’s disease patients with depression; NDPD, Parkinson’s disease patients without depression; HC, Healthy control.

**Supplementary Table 2 the head motion metrics (mean framewise displacement) in NDPD, DPD and HC groups**

| Groups | Average | SD | Average + 2SD |
| --- | --- | --- | --- |
| NDPD | 0.079 | 0.047 | 0.173 |
| DPD | 0.071 | 0.023 | 0.117 |
| HCs | 0.085 | 0.050 | 0.185 |

SD, standard deviation; DPD, Parkinson’s disease patients with depression; NDPD, Parkinson’s disease patients without depression; HC, Healthy control.

**Supplementary Table 3 The brain region with degree significantly correlated with HAMD scores in the DPD group**

| Region | Degree | r | P-value |
| --- | --- | --- | --- |
| IOG.R | 8.534±4.189 | 0.848 | **0.0001** |

The abbreviations of the 90 brain regions are given in Table 2.

DPD, Parkinson’s disease patients with depression; R, right hemisphere.

**Supplementary Table 4 The brain region with degree significantly correlated with HAMD scores in the NDPD group**

| Region | Degree | r | P-value |
| --- | --- | --- | --- |
| AMYG.L | 9.052±4.734 | -0.824 | **0.0002** |

The abbreviations of the 90 brain regions are given in Table 2.

NDPD, Parkinson’s disease patients without depression; L, left hemisphere.

**Supplementary Table 5 the network averages of the degree and betweenness in NDPD, DPD and HC groups**

|  | Groups | Average | SD | Average + SD |
| --- | --- | --- | --- | --- |
| degree | NDPD | 11.019 | 4.581 | 15.600 |
|  | DPD | 11.018 | 4.536 | 15.554 |
|  | HCs | 11.019 | 4.594 | 15.613 |
| betweenness | NDPD | 20.905 | 20.483 | 41.388 |
|  | DPD | 20.814 | 18.542 | 39.356 |
|  | HCs | 21.444 | 20.694 | 42.138 |

SD, standard deviation; DPD, Parkinson’s disease patients with depression; NDPD, Parkinson’s disease patients without depression; HC, Healthy control.

**Supplementary Table 6 The components of the subnetwork with decreased functional connectivity in the NDPD group compared with the HC group identified by NBS**

| Edge | Node 1 | Node 2 | T | P-value |
| --- | --- | --- | --- | --- |
| 1 | IFGoperc.L | OLF.R | -3.742 | **<0.001** |
| 2 | SMA.R | OLF.R | -4.947 | **<0.001** |
| 3 | SMA.R | REC.L | -3.982 | **<0.001** |
| 4 | SMA.R | REC.R | -3.658 | **<0.001** |
| 5 | OLF.R | PCL.R | -3.763 | **<0.001** |
| 6 | REC.L | PCL.R | -3.620 | **<0.001** |
| 7 | REC.R | PCL.L | -3.533 | **<0.001** |
| 8 | REC.R | PCL.R | -3.860 | **<0.001** |

The abbreviations of the 90 brain regions are given in Table 2.

DPD, Parkinson’s disease patients with depression. NDPD, Parkinson’s disease patients without depression; HC, Healthy control; L, left hemisphere; R, right hemisphere.

**Supplementary Table 7 The components of the subnetwork with decreased functional connectivity in the DPD group compared with the HC group identified by NBS**

| Edge | Node 1 | Node 2 | T | P-value |
| --- | --- | --- | --- | --- |
| 1 | ORBsup.R | ROL.R | -3.846 | **<0.001** |
| 2 | ORBsup.R | SMA.R | -3.822 | **<0.001** |
| 3 | ORBsup.R | DCG.L | -3.838 | **<0.001** |
| 4 | ORBsup.R | DCG.R | -3.770 | **<0.001** |
| 5 | ORBsup.R | FFG.R | -3.670 | **<0.001** |
| 6 | ORBsup.R | PCL.L | -4.227 | **<0.001** |
| 7 | ORBsup.R | PCL.R | -3.902 | **<0.001** |
| 8 | ORBsup.R | HES.R | -4.749 | **<0.001** |
| 9 | ORBsup.R | TPOsup.R | -3.584 | **<0.001** |
| 10 | ROL.R | REC.L | -3.968 | **<0.001** |
| 11 | SMA.R | OLF.R | -4.368 | **<0.001** |
| 12 | SMA.R | REC.L | -4.368 | **<0.001** |
| 13 | SMA.R | REC.R | -4.451 | **<0.001** |
| 14 | REC.L | AMYG.L | -3.521 | **<0.001** |
| 15 | REC.L | PCL.L | -3.594 | **<0.001** |
| 16 | REC.L | HES.R | -3.701 | **<0.001** |
| 17 | REC.L | STG.R | -3.624 | **<0.001** |
| 18 | REC.R | PCL.L | -3.985 | **<0.001** |
| 19 | CAL.R | LING.L | -4.078 | **<0.001** |
| 20 | CAL.R | LING.R | -4.495 | **<0.001** |
| 21 | CUN.R | LING.R | -4.447 | **<0.001** |
| 22 | CUN.R | IOG.R | -4.231 | **<0.001** |
| 23 | CUN.R | FFG.L | -3.534 | **<0.001** |
| 24 | CUN.R | FFG.R | -3.690 | **<0.001** |
| 25 | CUN.R | PCL.R | -3.534 | **<0.001** |
| 26 | LING.L | CAL.R | -4.078 | **<0.001** |
| 27 | LING.L | LING.R | -3.713 | **<0.001** |
| 28 | LING.R | SOG.L | -4.127 | **<0.001** |
| 29 | SOG.L | IOG.R | -4.498 | **<0.001** |

The abbreviations of the 90 brain regions are given in Table 2.

DPD, Parkinson’s disease patients with depression. NDPD, Parkinson’s disease patients without depression; HC, Healthy control; L, left hemisphere; R, right hemisphere.
